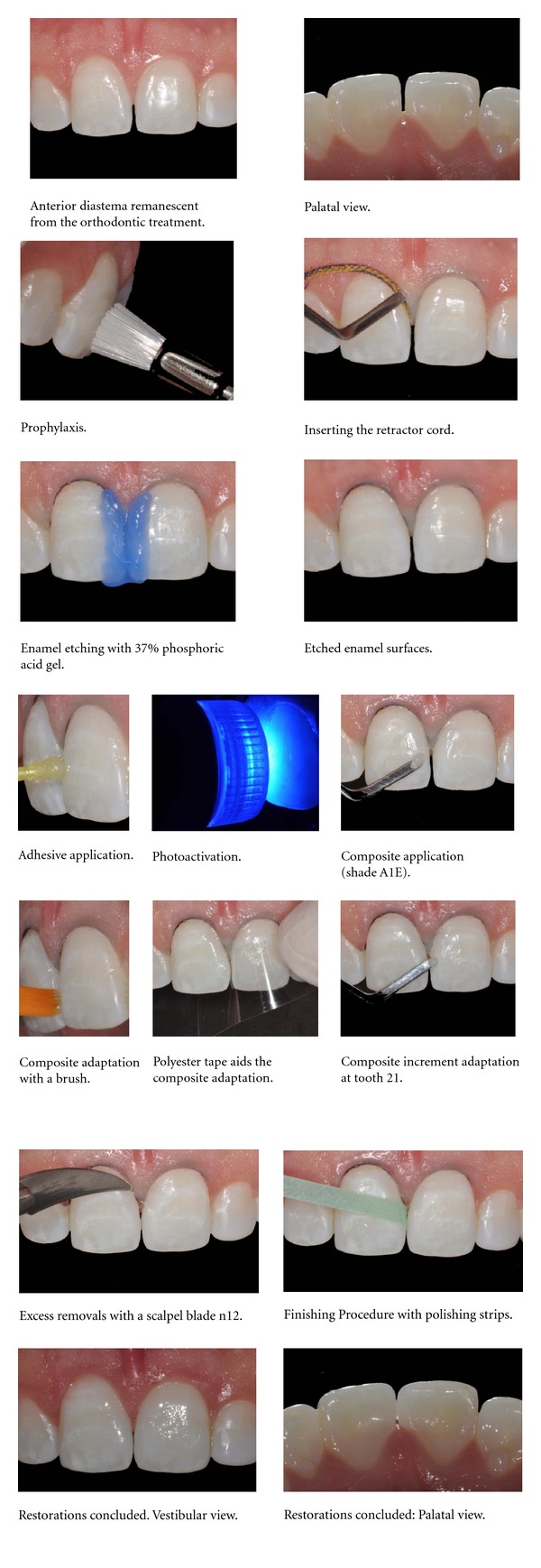# Nanofilled Composite Restorations with Different Adhesives Strategies: Clinical Cases

**DOI:** 10.1155/2012/969627

**Published:** 2012-08-02

**Authors:** Luana Dutra de Carvalho, Renata Gondo Machado, Guilherme Carpena Lopes, Mauro Caldeira de Andrada

**Affiliations:** Department of Dentistry, School of Dentistry, Federal University of Santa Catarina, Avenue Rubens de Arruda Ramos, 2354/201 Florianopolis, SC, Brazil

## Abstract

The esthetic procedures with composites are widely applied, both to posterior and anterior teeth to restore caries cavities, to replace failed restorations, or to make cosmetic procedures. The materials selected to each case may make the difference in the clinical result. This paper presents two clinical cases made with a nanofilled composite resin system used in different bond strategies. In the first, a wide posterior class I restoration, the self-etching strategy was used. The second, an esthetic anterior restoration, was made using the prior etching with phosphoric acid and a hydrophobic adhesive.

## 1. Introduction

The adhesive systems were developed in order to ensure the composite bonds to enamel and dentin. In 1955, Buonocore suggested that the enamel treatment with an acid would cause the formation of microporosities on its surface, responsible for the acrylic resins penetration, promoting an effective mechanical connection [1]. Since then, the enamel adhesion was considered a predictable procedure. The greatest difficulty was effective bond to dentin. Therefore, over the years, the adhesive systems used for this union have changed the composition and new formulations have been proposed with the aim of achieving a more effective and durable adhesion, trying to reduce the number of application steps.

Currently, the adhesives may be classified according to the way they interact with the smear layer [2]. They are divided into subcategories according to the number of application steps: (1) total etching a systems adhesives: including the etching step with 30–40% phosphoric acid applied simultaneously at enamel and dentin. That removes the smear layer and superficial hydroxyapatite to receive the primer/adhesive application. This category is subdivided into

- Three-step total-etch (acid + primer + adhesive);
- two-step total-etch (acid + primer/adhesive).

(2) Self-etching adhesive systems do not require the etching step and include an acidic monomers solution, which is applied without requiring rinsing, making the smear layer permeable, without the complete remove. This category is subdivided into

- two-step self-etch (acidic primer + adhesive);
- single-step self-etch (only one solution, i.e., “all in one”).

The introduction of self-etching systems modified the traditional adhesion concept, eliminating the need for an additional etching step. However, with these systems, it is necessary to use an acidic monomer which acts as etch and primer. After that, a hydrophobic adhesive layer may be used to cover the primer.

The adhesive dentistry tendency is to simplify procedures. The multiple steps of adhesive systems have been replaced by simplified systems, apparently easier to use and, in theory, systems that require a shorter application time [3].

Regarding the composites, from the time they were developed in the 60s, a significant evolution occurred. An improvement in these materials mechanical properties allows the application even in posterior teeth. Until recently, the most important changes have involved the reinforcing filler, which has been purposely reduced in size to produce materials that are more easily and effectively polished and demonstrate greater wear resistance. The latter was especially necessary for materials used in posterior applications, but the former has been important for restorations in all areas of the mouth. In addition, the modern systems have different degrees of translucency that enables the reproduction of the enamel, dentin, and the natural tooth optical effects [4].

The cases reported describes two different adhesive system applications, according to their specifications. A nanofilled composite system wasselected to perform both restorations.

**Case 1**

Composite resin restoration on tooth 36 with the use of the one-bottle self-etching adhesive Adper Easy One and the composite resins Filtek Z350XT (3M/ESPE).

A 20-year-old female patient, with tooth 36 requiring restoration replacement due to marginal leakage and caries lesion under the restoration.

**Figure 1:**
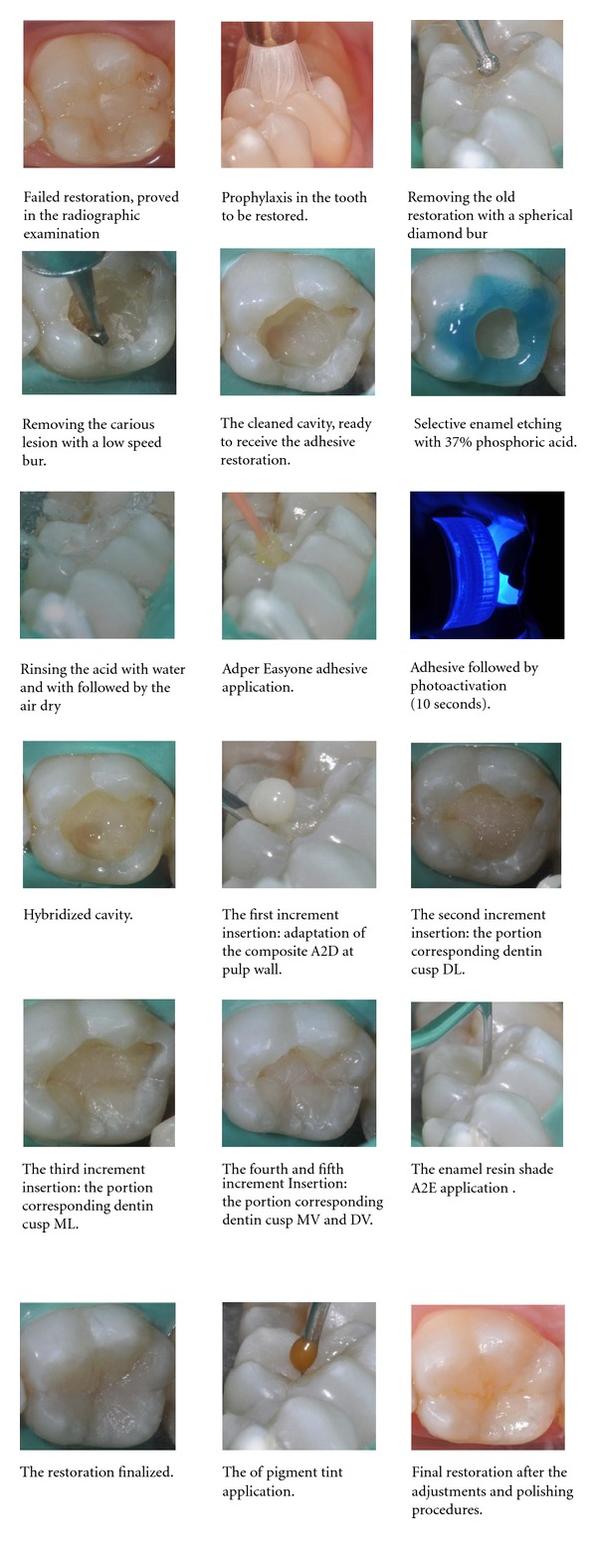


After clinical and radiographic examination, the restorative procedure was performed as the follow sequence (Figure 1).

**Case 2**

Composite resin restoration for anterior diastema closure with the use of an adhesive system with phosphoric acid prior. The Scotchbond Multipurpose and composite resin Filtek Z350XT (3M/ESPE) were used.

A 28-year-old male patient presents a recurrent anterior diastema after the orthodontic treatment. As he did not accept orthodontic retreatment, the option was closure with composite resin. After clinical and radiographic examination, the color was selected and the esthetic restorations were confectioned as the following steps (Figure 2).

In both cases, each composite increment was photoactivated for 40 seconds, with a LED device (Flash-lite-discus dental). The color assessment and the polishing procedures were performed 24 hours after the restorative procedures.

## 2. Discussion

The clinical cases reported demonstrate different ways of adhesive systems interaction with the dental tissues. Regarding the self-etching, the main advantage of that is the ability to demineralize the dentin simultaneously to the adhesive monomer penetration. That is interesting in deep dentin cavities, close to pulp tissue [5, 6]. This supposedly would generate a lower postoperative sensitivity [7]. However, there is a lower monomers penetration capacity of these acidic monomers trough the enamel [6]. This would create a weak link, with less retention and greater possibility of microleakage, represented clinically by a higher marginal staining [8, 9]. Due to this, the adhesion to enamel is better when the etching is performed with phosphoric acid (step performed in both cases). The phosphoric acid etching prior to the adhesive application allows a more efficient and durable bond [10]. The demineralization process enamel selectively dissolves the enamel rods, creating microporosities which are readily penetrated, even by ordinary hydrophobic bonding agents, by capillary attraction. Upon polymerization, this micromechanical interlocking of tiny resin tags within the acid-etched enamel surface still provides the best achievable bond to the dental substrate [11]. In the clinical case, the Bond (step 3) from the ScotchBond Multipurpose was used. This option was chosen because it is a hydrophobic and solvent-free adhesive. This was possible because the restoration area was exclusively in the enamel. The absence of solvent and hydrophilic monomers in the composition would produce an improved union.

Although there are different forms of hybridization, one of the goals of the manufacturers is to combine all the functions necessary for a good bond with the lowest number of clinical steps. However, by placing various components with different functions in the same vial, the bond ability can be impaired by the high hydrophilicity of the monomers and the high solvent concentration is required to maintain the stability. By the difficulty of mixing the components, water is needed to ionize the medium and allow activity self-etching [12, 13]. This may generate an instability of the cured adhesive layer with an increased permeability and a possible long-term bond deterioration. The single-step self-etching adhesives are not better than the multiple steps. Their bond strength tends to be lower and the application procedures are not always faster [14].

Regarding the composites, nanofilled were selected for the clinical cases reported. These are an actual and complete composites option. The nanofill and nanohybrid materials represent the state of the art in terms of filler formulation [15, 16]. Most recently, these composites demonstrated the advantages of previous composite generations, such as strength, low wear, and polishability, but without many of the limitations [17]. Furthermore, the selected system has different shades, allowing the natural dental tissue reproduction in an efficient and durable way, using the stratification technique [18, 19].

The relevance of using products with scientific evidence makes the results much safer. Therefore, irrespective of the selected products, that should be from suitable manufacturers. Finally, with the materials available on the market today, with the efficacy proved by researchers from different laboratories around the world, we can rebuild lost structures with esthetic restorations, effective bond, and patients' satisfaction.

## Figures and Tables

**Figure 2:**